# Altered Waste Disposal System in Aging and Alzheimer’s Disease: Focus on Astrocytic Aquaporin-4

**DOI:** 10.3389/fphar.2019.01656

**Published:** 2020-01-29

**Authors:** Marta Valenza, Roberta Facchinetti, Luca Steardo, Caterina Scuderi

**Affiliations:** ^1^ Department Physiology and Pharmacology “V. Erspamer”, Sapienza University of Rome, Rome, Italy; ^2^ Epitech Group SpA, Saccolongo, Italy; ^3^ Università Telematica Giustino Fortunato, Benevento, Italy

**Keywords:** aquaporin-4, aging, Alzheimer’s disease, astrocytes, glymphatic system, brain clearance, perivascular space

## Abstract

Among the diverse cell types included in the general population named glia, astrocytes emerge as being the focus of a growing body of research aimed at characterizing their heterogeneous and complex functions. Alterations of both their morphology and activities have been linked to a variety of neurological diseases. One crucial physiological need satisfied by astrocytes is the cleansing of the cerebral tissue from waste molecules. Several data demonstrate that aquaporin-4 (AQP-4), a protein expressed by astrocytes, is crucially important for facilitating the removal of waste products from the brain. Aquaporins are water channels found in all district of the human organism and the most abundant isoform in the brain is AQP-4. This protein is involved in a myriad of astrocytic activities, including calcium signal transduction, potassium buffering, synaptic plasticity, astrocyte migration, glial scar formation and neuroinflammation. The highest density of AQP-4 is found at the astrocytic domains closest to blood vessels, the endfeet that envelop brain vessels, with low to zero expression in other astrocytic membrane regions. Increased AQP-4 expression and loss of polarization have recently been documented in altered physiological conditions. Here we review the latest findings related to aging and Alzheimer’s disease (AD) on this topic, as well as the available knowledge on pharmacological tools to target AQP-4.

## Introduction

During the past 15 years, glial cells have gained noticeable attention, as their complex and heterogeneous functions were progressively getting discovered and understood. Glial cells have been recognized as essential supportive cells for neurons with a variety of specific and crucial homeostatic functions, including, but not limited to, uptake and release of chemical transmitters (Allen and Barres, 2009). For example, a growing body of literature demonstrates that synaptic function and plasticity require not just the presynaptic and postsynaptic neurons, but also the presence of glial cells, specifically astrocytes, Schwann cells, and microglia (Araque et al., 1999) with the contribution of the extracellular matrix too, forming a multi-partite structure referred as synaptic cradle (Dityatev and Rusakov, 2011; Verkhratsky and Nedergaard, 2014; Pekny et al., 2016; Verkhratsky and Nedergaard, 2018).

Among the diverse cell types included in the general population named glia, astrocytes emerge as being the focus of a growing body of research aimed at characterizing their heterogeneous and complex functions. Indeed, alterations of both their morphology and activities have been linked to a variety of neurological disorders and diseases (Scuderi et al., 2013; Scuderi et al., 2018b). Multiple and disparate changes occur in astrocytes (e.g., from hypertrophy to atrophy, from proliferation to cell death) in a highly heterogeneous and complex way, both context-dependent and disease-specific. Astroglial pathological modifications are driven by different signaling mechanisms and produce diverse responses from adaptive to maladaptive, and further they may change along the course of a disease (Sofroniew, 2014; Pekny et al., 2016; Verkhratsky et al., 2017).

One, out of many, crucial physiological need satisfied by astrocytes is the cleansing of the cerebral tissue from waste molecules. Indeed, without a waste disposal system, the brain would accumulate unwanted molecules that would interfere with its optimal functioning. Such cleansing system has been the topic of intense research and debates among scientists. In 2012 the original view of waste products disposed by diffusion was challenged by the publication of a research paper describing a water and solute clearance system regulated by astrocytes (Iliff et al., 2012). The authors indeed named it glymphatic system to underline the crucial role of glial cells. Experiments were carried out in living mice, injecting fluorescent tracers into the subarachnoid space of the brains, and then imaging their real-time movement using two-photon microscopy. Results suggested that the cerebrospinal fluid (CSF, mimicked by the tracers) moves by convective flow along the perivascular space between a vessel and the endfeet of astrocytes escheating the vasculature. The fluid penetrates the extracellular space of the parenchyma from the perivascular space as the artery branches into arterioles and capillaries. At this level, the CSF mixes with the interstitial fluid filling up of metabolic waste, moving by diffusion (Holter et al., 2017) toward the perivascular space of venules and capillaries to ultimately reach the lymphatic vessels (Louveau et al., 2015), which drain the molecules absorbed from the dural meninges to the cervical lymph nodes (Aspelund et al., 2015). This system was found dependent on aquaporin-4 (AQP-4), a bidirectional water channel highly expressed by astrocytes, since deletion of *Aqp-4* gene in mice severely reduced (nearly 70%) clearance from the brain (Iliff et al., 2012; Mestre et al., 2018). Authors then conclude that AQP-4 facilitates convective flow out of the periarterial space and into the interstitial space (Iliff et al., 2012; Nedergaard, 2013).

Thirteen aquaporins have been identified so far and, among them, the AQP-4, isolated from rat brain in 1994 (Hasegawa et al., 1994; Jung et al., 1994), is recognized as the most abundant water channel of the central nervous system (CNS). It is expressed by glial cells, specifically by astrocytes and ependymal cells, mostly in regions close to vessels throughout the CNS, including the spinal cord, and the cerebellum (Jung et al., 1994; Frigeri et al., 1995). Two isoforms have been identified in humans, that are AQP-4-M1 and AQP-4-M23 (Sorani et al., 2008a; Sorani et al., 2008b). Nielsen and collaborators were the firsts to describe that astrocytes express polarized AQP-4, such that the higher density of the channel is found at domains closest to blood vessels and the pia mater, with low to zero expression in other astrocytic membrane regions, except for some synapses (Nielsen et al., 1997).

The presence of the glymphatic disposal system in the human brain has not been fully demonstrated yet, although some evidence concurs to confirm it (Eide and Ringstad, 2015; Taoka et al., 2017; Rasmussen et al., 2018). Despite these, not all scientists believe that such glymphatic waste system actually exists, at least as presented by Iliff et al. (2012) because of some inconsistent findings suggesting that solute transport does not depend on the astrocytic AQP-4 (Smith et al., 2017; Iliff and Simon, 2019; Smith and Verkman, 2019). Debates are ongoing about the type of flow supporting the clearance system, as it is pressure-driven convective flow (generated by pulsation of arteries and collapse and inflation of veins) (Iliff et al., 2013; Ray et al., 2019), or diffusive down to gradient (Asgari et al., 2016; Smith et al., 2017; Smith and Verkman, 2018). Despite this, evidence demonstrates that AQP-4 deletion impairs blood-brain interface permeability to water (Papadopoulos and Verkman, 2005).

**Figure 1:**
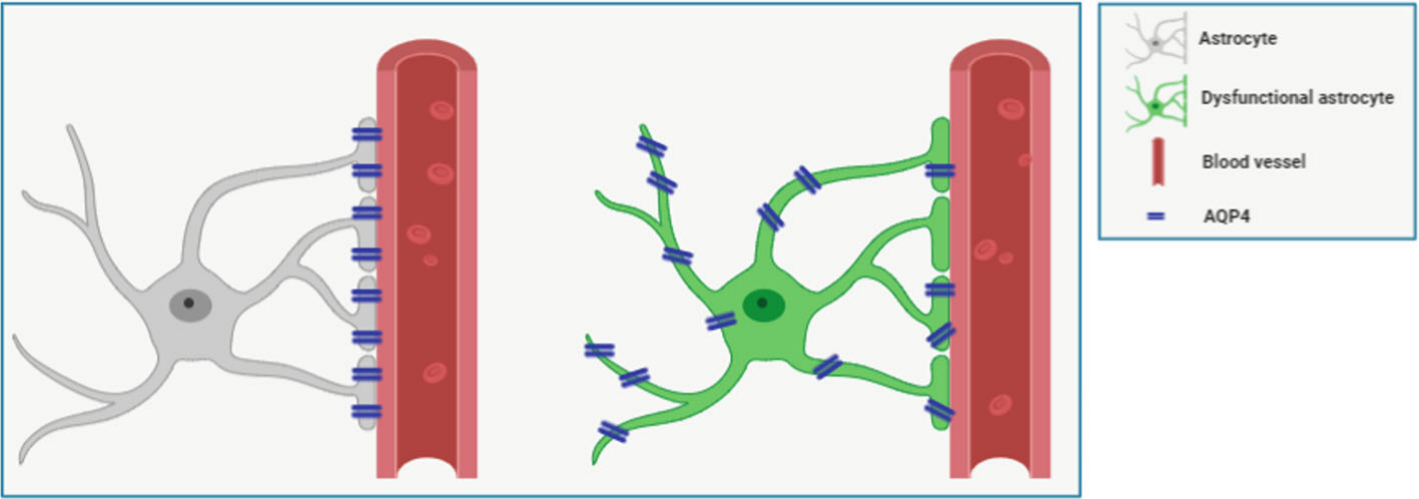
Figure shows representative schemes for expression and polarization/localization of AQP-4 in healthy (*left*) and dysfunctional (*right*) perivascular astrocyte. Astrocytes processes wrap the vessel forming a sheath around it. Cerebrospinal fluid (CSF) flows in the perivascular space created around the vessel. The astrocytic water channel AQP-4 is polarized, as it is densely expressed by astrocytes almost exclusively at the endfeet, in direct contact with the perivascular space, where it facilitates the interchanges of water. In aging and some pathological conditions, such as Alzheimer’s disease (AD), AQP-4 loses its polarization in reactive astrocytes and it is found diffusively expressed. Also, higher AQP-4 expression has been documented in Parkinson’s disease, cerebral ischemia, amyotrophic lateral sclerosis, and other neurological diseases (for review see Xiao and Hu, 2014; Mader and Brimberg, 2019).

Despite the ongoing scientific debates, some new findings have been collected during the past 5 years valuing the notion that specific AQP-4 localization in astrocytes and its expression might be crucial aspects in physiological and pathological conditions (**Figure 1**). Here we review the latest findings related to aging and AD on this topic, as well as the available knowledge on pharmacological tools to target AQP-4. However, AQP-4 is involved in a myriad of astrocytic activities, including calcium signal transduction (Thrane et al., 2011), potassium buffering (Jin et al., 2013), synaptic plasticity (Fan et al., 2005; Ding et al., 2007; Zeng et al., 2007), astrocyte migration (Saadoun et al., 2005; Auguste et al., 2007), glial scar formation (Saadoun et al., 2005; Wu et al., 2014), and neuroinflammation (Li et al., 2011) (for extensive review refer to Xiao and Hu, 2014; Hubbard et al., 2018; Mader and Brimberg, 2019).

### AQP-4 in Aging and Alzheimer’s Disease

Aging is the greatest risk factor for developing dementia and Alzheimer’s disease (AD). Aging is a process that involves the whole organism, including the clearance system of the brain. It is often associated with shorter duration of sleep time (Wolkove et al., 2007), which is the period of activity of the aforementioned cerebral waste disposal system (Xie et al., 2013). *Aqp-4* gene expression has been found increased in cerebral and cerebellar cortices of aged (17-month-old) mice compared to their adult counterpart (Gupta and Kanungo, 2013). Similarly, age-dependent raise in AQP-4 expression has been reported in the hippocampal CA1 region of 12-month-old compared to 6-month-old 3×Tg-AD mice, a triple transgenic model of AD, irrespective of genotype (Bronzuoli et al., 2019). In accordance, Zeppenfeld et al., reported in 2017 that altered AQP-4 immunostaining was associated with increasing age in *post-mortem* human cortices. Therefore, it can be hypothesized that the upregulation of astrocytic AQP-4 responds to a physiological need for compensating general astrocytes morphological or functional alterations known to occur both in rodents and human *post-mortem* aged brains (Hoozemans et al., 2011; Bronzuoli et al., 2019). However, this hypothesis needs further direct demonstrations.

Aged brains show also altered AQP-4 localization (Zeppenfeld et al., 2017). Indeed, a study from the Nedergaard group demonstrated increased perivascular GFAP in aged (18 months) compared to young (2–3 months) C57BL/6 mice, coupled with a significant, but modest, loss of perivascular localization (Kress et al., 2014). A loss of vascular localization of AQP-4 has been demonstrated in old (24-months) compared to young (6-months) TgSwDI mice, which develop age-dependent accumulation in amyloid, together with general reactive gliosis, as shown by increased number of GFAP-positive astrocytes and Iba 1-positive microglia (Duncombe et al., 2017). Preservation of perivascular localization of AQP-4 in aged human individuals was predictive of preserved cognitive abilities (Zeppenfeld et al., 2017). Additionally, the arterial pulsating force was lower as well as the rate of clearance of the tracer injected into the brains was slower in aged compared to young C57BL/6 mice (Kress et al., 2014).

Measurements of beta-amyloid (Aβ) deposition in human by positron emission tomography (PET) show that Aβ begins to abnormally deposit within the brain between age 40 and 50, thus far before clinical symptoms (Villemagne et al., 2013). This stage of the disease is termed preclinical or prodromal AD; it is characterized by patients having no symptoms of the disease yet, and only few molecular alterations have begun to appear (Hyman et al., 2012). Oxidative stress, as well as signs of neuroinflammation and reactive astrocytes, have been documented at early stages of the disease, before the appearance of massive Aβ deposition and tau hyper-phosphorylation (Zhu et al., 2004a; Zhu et al., 2004b; Jack et al., 2010; Rodriguez-Vieitez and Nordberg, 2018). In absence of neuronal atrophy, a premature presence of reactive astrogliosis can be detected in animal models of AD, as in 6-month-old 3×Tg-AD mice (age that corresponds to a mild stage of pathology). A study using a novel non-invasive magnetic resonance imaging protocol reports lower water influx into the CSF of mice expressing high senile plaque density (APP/PS1 mice) compared to their wild-type counterpart (Igarashi et al., 2014a), similar to what seen in AQP-4 knock-out mice (Igarashi et al., 2014b). AQP-4 knock-out mice show reduced (−50%) intracerebrally infused Aβ clearance compared with wild-type littermates (Iliff et al., 2012). The association of AQP-4 deletion in APP/PS1 mice brought to a significant increase of both soluble and insoluble Aβ in the brain, without affecting synthesis or degradation of the protein (Xu et al., 2015). Moreover, bidirectional relationship between sleep and AD has been reported, such that patients with AD experience sleep disturbances as well as poor sleep predisposes to AD (Ju et al., 2014). Indeed, brain waste products, such excessive Aβ and tau, are cleared during sleep time (Xie et al., 2013; Shokri-Kojori et al., 2018). Based on this, a recent report investigated the association of single-nucleotide polymorphisms (SNPs) in *Aqp-4* gene with sleep latency, duration, and amount of radiolabeled Aβ imaged through PET scans carried out in healthy volunteers >60 years old. They found one SNP associated with poor sleep quality, and two SNPs associated with short sleep duration and consequent higher Aβ burden. In contrast, one SNP, the rs2339214, was associated with higher Aβ and also longer sleep duration (Rainey-Smith et al., 2018). All these accumulating evidence suggests that deposits of Aβ and tau are consequences of impaired clearance, rather than of increased production (Benveniste et al., 2019).

Burfeind and collaborators identified five SNPs in the *Aqp-4* gene and analyzed their possible association with cognitive decline exclusively in AD patients. Their results identified two *Aqp-4* SNPs associated with rapid, and two with slow, cognitive decline (Burfeind et al., 2017). Another report from the same group studied the association between perivascular AQP-4 localization and its expression levels with AD pathology in humans, showing for the first time that total AQP-4 expression was increased in the AD cortex compared to cognitively intact subjects, both young and aged. The raise was correlated with Aβ deposits. Additionally, loss of perivascular AQP-4 was associated with AD Braak stage and density of Aβ plaques (Zeppenfeld et al., 2017). Ten years before, increased expression of AQP-1, but not AQP-4, was reported in the frontal cortex of patients with early AD stage (Perez et al., 2007). AQP-4 was found highly diffused in the parenchyma of *post-mortem* human AD brains and of a mouse model of AD (5xFAD), with particular localization near Aβ plaques rather than near vasculature (Smith et al., 2019), supporting the hypothesis that a change in AQP-4 localization might be a crucial aspect in AD neuropathology. Interestingly, since 5xFAD mice showed increased neuronal Aβ, they propose that AQP-4 peri-plaques localization might be a defense mechanism to counteract Aβ deposition (Smith et al., 2019). However, further studies are needed to demonstrate this novel and intriguing hypothesis. Anyway, the cited evidence supports the idea that several alterations, including control of water, ions and solute clearance, occur in aging and early stages of AD.

### Pharmacological Tools Targeting AQP-4

Despite the massive preclinical and clinical efforts, no effective treatments are currently available for patients with AD. Recent evidence concurs that the best time for intervention is when the disease is not fully overt. This preclinical phase of the disease is difficult to diagnose because, at present, there are no specific biomarkers able to reliably and timely detect it. Disappointing results of the latest clinical trials has prompted researchers to rethink possible pharmaceutical targets and therapeutic approaches, including targeting AQP-4. However, malfunction of the brain cleansing system because of aging brings to waste piling up, including proteins as Aβ and tau. Therefore, astrocytic AQP-4 seems to represent a possible pharmacological candidate to be targeted in AD at its earliest stage, before abnormal protein accumulation and neurodegeneration occur. So far, some molecules have been tested for activity to AQP-4, but none in *in vitro* or *in vivo* models of AD (Lan et al., 2016; Tradtrantip et al., 2017). Some phytocompounds with antioxidant properties have shown to be active on AQP-4. Among them, pinocembrin, a flavonoid contained in propolis, seems to be able to downregulate AQP-4 expression in a rodent model of focal cerebral ischemia (Gao et al., 2010); curcumin treatment reduced hypoxia-hypercapnia-induced brain edema by downregulating the messenger RNA (mRNA) expression levels of AQP-4 in rats (Yu et al., 2016) and dampening AQP-4 and GFAP overexpression in a rat model of acute spinal cord injury (Nesic et al., 2010). Similar results were published with epigallocatechin gallate treatment, an essential ingredient of green tea (Ge et al., 2013). Acute administration of carvacrol, a terpenoid, dose-dependently attenuates brain edema induced by cerebral hemorrhage in mice by downregulating brain *Aqp4* gene and protein expression, likely reducing astrocyte swelling (Nesic et al., 2010). Preliminary studies in our laboratory suggest that *in vivo* chronic treatment of 3×Tg-AD mice and their wild-type counterpart with the ALIAmide palmitoylethanolamide (PEA) is able to reduce the upregulated expression of hippocampal AQP-4 selectively in AD-like mice. Numerous evidence demonstrates the anti-inflammatory and neuroprotective properties of PEA (Scuderi et al., 2012; Scuderi et al., 2014; Skaper et al., 2015), and we have recently demonstrated *in vivo* the efficacy of a formulation of ultramicronized PEA (um-PEA) in reducing several AD-like molecular and behavioral signs in 3×Tg-AD mice (Bronzuoli et al., 2018; Scuderi et al., 2018a). However, further studies are needed to verify the effects of formulations containing PEA on AQP-4 expression and functions.

Interestingly, it has recently been reported that atorvastatin, already in use in the clinical setting as lipid-lowering drug, may prevent ischemic brain edema through downregulation of astrocytic AQP-4 expression in rats. Authors proposed a mechanism involving the attenuation of p38-MAPK signaling (Cheng et al., 2018). Similarly, 2-(nicotinamide)-1,3,4-thiadiazole (TGN-020) was shown to act as a potent AQP-4 inhibitor in a rodent model of ischemia (Pirici et al., 2018; Catalin et al., 2018). A Japanese herbal compound named Goreisan was able to reduce edema in an *in vivo* model of hypoxic-ischemic encephalopathy by reducing the lesion-induced upregulation of AQP-4 protein expression, and ameliorating the rat survival rate compared to the control group (Nakano et al., 2018). Similarly, in a rat model of traumatic brain injury (TBI), acute administration of the hormone ghrelin was able to prevent post-TBI upregulation of AQP-4 expression (Lopez et al., 2012). Chronic treatment with dabigatran etexilate, a thrombin inhibitor, showed an indirect effect on AQP-4, preventing its misplacement found in TgCRND8 mice, a mouse model of AD (Cortes-Canteli et al., 2019). Thus, converging evidence demonstrates that targeting AQP-4 seems to be a promising pharmacological approach in several brain pathologies. For example in major depressive disorder there is a clear reduction in the coverage of blood vessels by AQP-4-positive astrocyte endfeet (Rajkowska et al., 2013). Intriguingly, Di Benedetto and collaborators found that AQP-4 is necessary to mediate fluoxetine-induced growth of astrocytic processes in rats (Di Benedetto et al., 2016).

New AQP-4 partial antagonists have been discovered by library screening by Aeromics, Inc. (OH, USA). The drug AER-270, and its prodrug with enhanced solubility AER-271, have shown beneficial results on brain edema in two different model of cerebral injury in rats, reducing swelling and behavioral neurological damage (Farr et al., 2019). Since AQP-4 was found up-regulated in the aging brain, and mislocalized in AD, it would be interesting to test the hypothesis that treatment with AQP-4 modulator may slower brain senescence process and prevent neurological deficit through a fine regulation of this water channel. However, the effect of therapeutic interventions targeting AQP-4 will depend on the balance between the beneficial increased water clearance and deleterious effects on astrocytic morphological changes. Since not all pathological conditions are associated with impaired blood brain barrier (BBB), AQP-4-targeting drug should be able to cross an intact BBB, as for example in prodromal stages of AD. However, reaching this perfect balance between maximum benefit and limited toxicity depends on future further understanding of the biology of AQP-4.

## Author Contributions

Conceptualization: MV, CS. Data curation: MV, RF, CS. Draft preparation: MV. Mini review final editing: MV, RF, LS, CS.

## Funding

This work was supported by the Italian Ministry of Instruction, University and Research (MIUR) (PRIN 2015 grant n. 2015KP7T2Y_002) and the SAPIENZA University of Rome (grant n. MA116154CD981DAE) to CS.

## Conflict of Interest

MV discloses a collaboration with Epitech Group SpA, which had no role in the conceptualization and writing of the mini review, and in the decision to submit the paper for publication.

The remaining authors declare that the research was conducted in the absence of any commercial or financial relationships that could be construed as a potential conflict of interest.

The reviewer AV declared a past co-authorship with several of the authors RF, CS, LS to the handling editor.
